# Primary Vaginal Non-Hodgkin Lymphoma

**DOI:** 10.7759/cureus.3713

**Published:** 2018-12-11

**Authors:** Pamela Contreras-Chavez, Rolig Aliaga, Mohammed Samee, Andrea Anampa-Guzmán

**Affiliations:** 1 Internal Medicine, Advocate Illinois Masonic Medical Center, Chicago, USA; 2 Oncology, Hospital Nacional Arzobispo Loayza, Lima, PER; 3 Internal Medicine, Universidad Nacional Mayor De San Marcos, Lima, PER

**Keywords:** hematologic malignancy, vagina

## Abstract

Non-Hodgkin lymphoma (NHL) is a type of blood cancer and 25% of NHL patients present with a primary extranodal tumor. Primary NHL of the vagina is extremely rare with only a few cases reported. We present the case of a 79-year-old, grand multipara, Peruvian woman with an eleven-month history of symptoms of pelvic and vaginal pain and vaginal bleeding. Vaginal examination revealed an exophytic tumor that involved the labia, right vaginal wall, cervix, parametrium, and pelvic bone. A computed tomography (CT) image showed a 10 x 10 x 9-cm solid tumor in the pelvic cavity with irregular edges in the pelvic floor, parametrium, and the perineal soft tissues. Bilateral pelvic and inguinal adenomegalies were found. No signs of metastases were found. Marrow and bone biopsy studies were negative. The diagnosis was NHL type diffuse large B-cell lymphoma (DLBCL) of vagina stage one. The patient was treated with six courses of CHOP-R (cyclophosphamide, doxorubicin, vincristine, prednisone, and rituximab). She showed a complete response and remained in remission in follow-up control visits. NHL of the female genital tract is rare. CHOP-R is the first line of treatment for this type of cancer. However, less is known about the follow-up protocol and relapse management. Vaginal lymphoma has an extremely low prevalence, and collaborative studies are required to study the same.

## Introduction

Non-Hodgkin lymphoma (NHL) is a group of lymphoid malignancies, which typically develop in the lymph nodes but may occur in almost any tissue. Twenty-five percent of NHL patients present with a primary extra-nodal tumor [1]. Ninety percent of the NHL cases are of B-cell origin and 10% derived from T cells or natural killer (NK) cells. NHLs have a different range of histological appearances and clinical features at presentation. Diffuse large B-cell lymphoma (DLBCL) is the most common subtype of NHL, representing about a third of cases [2].

Hematologic malignancies rarely present as a primary gynecologic problem. They usually occur as a result of the systemic dissemination of the tumor cells rather than the primary tumor. Extranodal NHL often involves the gastrointestinal tract and the bone marrow. Less than 0.5% of all extranodal NHLs involves the female genital tract [3]. Primary NHLs of the vagina is extremely rare with only a few cases reported [4-5].

## Case presentation

**Figure 1:**
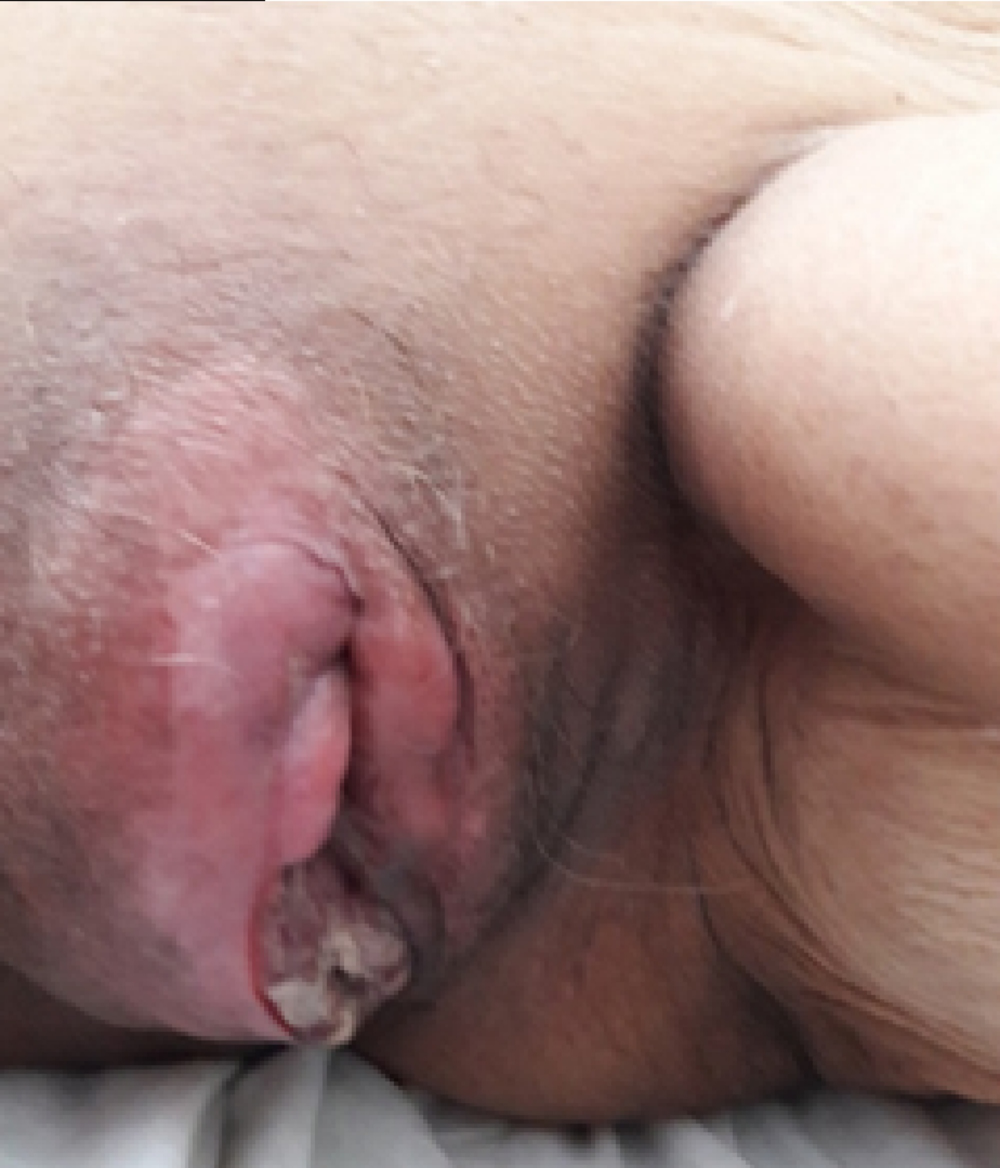
Pre-treatment

A 79-year-old Peruvian woman presented with 10 months of vaginal bleeding and a vaginal tumor that was exophytic involving the cervix and extending to the right vaginal wall. There was a right tumoral mass that involved the sub-epithelium of the labia to the pelvic bone. The right parametrium was invaded until the pelvic bone. Computerized tomography (CT) scans showed a large solid tumor mass (10 x 10 x 9 cm) in the pelvic cavity with irregular edges that infiltrated the pelvic floor, parametrium, and the perineal soft tissues (Figure 1).

Hematoxylin-eosin showed a proliferation of the hyperchromatic cells with nuclear pleomorphism associated with an apparently red cell neoplasm. Immunohistochemistry was negative for Pankeratin, Melan-A, S100, and CD3. CD20, BCL2, BCL6, CD10, MU-1, and C-MYC were positive. Ki-67 was positive and over-expressed in 70% of the cells. Chest CT showed an interstitial reticular pattern and no signs of nodules and lung masses. Bone marrow and bone biopsy were negative. The final diagnosis was vaginal NHL of large B cells, with the primary central germinal phenomenon, stage IE with a bulky mass.

**Figure 2:**
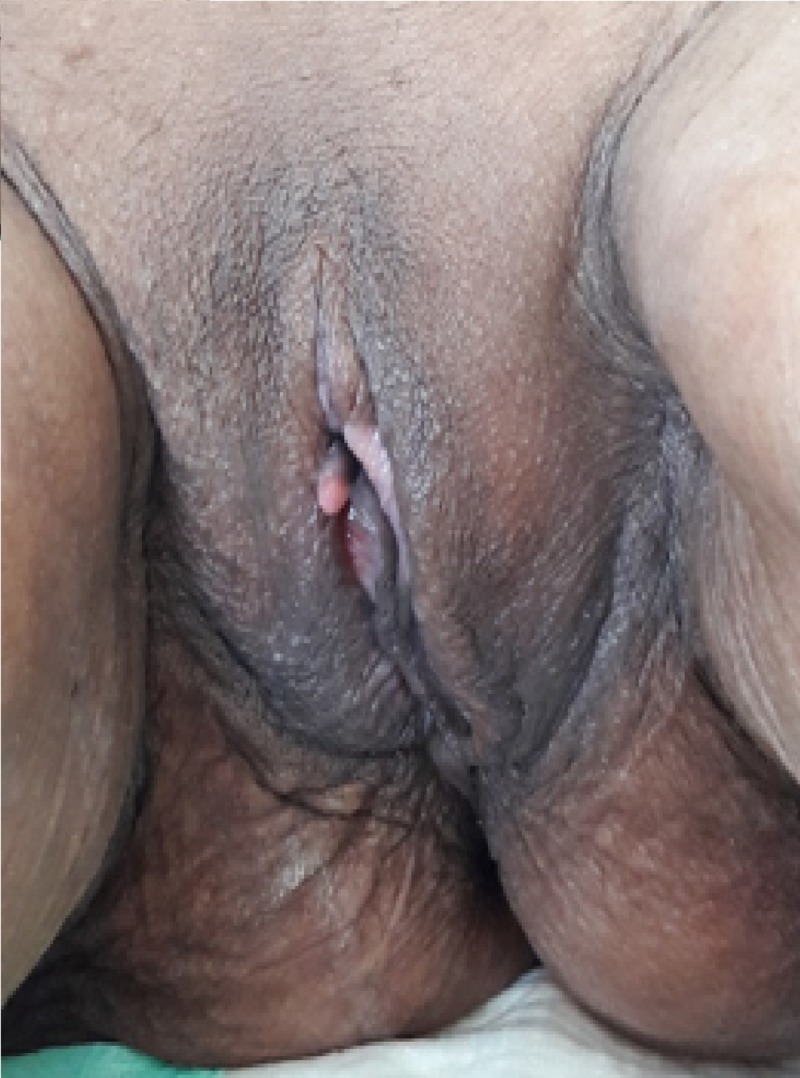
Post-treatment

The initial treatment was CHOP-R (cyclophosphamide, doxorubicin, vincristine, and prednisone, plus rituximab). The patient completed six courses of CHOP-R chemotherapy. The last clinical evaluation showed a complete clinical response (Figure 2). The patient was under control.

## Discussion

NHLs are a large group of lymphoid neoplasms, with different morphologic, immunophenotypic, and genetic characteristics. NHL of the female genital tract is rare [6]. The primary NHLs of the female genital tract involve the cervix in the majority of cases. The second most common sites are the ovaries and the uterine corpus [7]. The largest series of malignant lymphomas involve the female genital organs; primary vaginal NHLs account for less than <0.1% [8].

Primary vaginal lymphomas have been reported in women of age 26 to 66 years [9]. This lymphoma presents most often with a vaginal mass. The tumor is usually infiltrative with a thick vaginal wall [7]. Like in our case, the most common type of primary vaginal NHL is DLBCL [6]. Patients may also complain of abnormal vaginal bleeding, mass at the introitus, dyspareunia, vaginal discharge, and irritative urinary symptoms [9-10]. The Lugano classification is the current staging system for patients with NHL [11]. Our case was stage IE.

Due to its rarity, there is no established treatment for primary NHL of the vagina. The first line of treatment for NHL is chemotherapy since NHLs are extremely responsive to it [12]. The treatment for limited-stage DLBCL is CHOP-R, including cyclophosphamide, doxorubicin, vincristine, prednisone, and rituximab, a monoclonal antibody directed against the CD20 antigen. Radiation therapy can either be added or not at all [13]. The primary pelvic lymphomas have a five-year survival rate of 80% to 90% if the diagnosis was made early and therapy was adequate [14]. Our case had a good outcome similar to these statistics.

## Conclusions

Vaginal lymphoma has an extremely low prevalence. CHOP-R is the first line of treatment for this type of cancer. However, little is known about the follow-up protocol and relapse management. More collaborative studies are required to study vaginal lymphoma.
